# Co-design and mixed methods evaluation of an interdisciplinary digital resource for undergraduate health profession students to improve the prevention, recognition, and management of delirium in Ireland: a study protocol

**DOI:** 10.1186/s12909-024-05468-1

**Published:** 2024-04-30

**Authors:** Lana Cook, Alice Coffey, Christine Brown Wilson, Pauline Boland, Patrick Stark, Margaret Graham, James McMahon, Dympna Tuohy, Heather E Barry, Jill Murphy, Matt Birch, Audrey Tierney, Tara Anderson, Arlene McCurtin, Emma Cunningham, Geoffrey M. Curran, Gary Mitchell

**Affiliations:** 1 School of Nursing and Midwifery, Queen’s University Belfast, Belfast, UK; 2 Department of Nursing and Midwifery, University of Limerick, Limerick, Ireland; 3 School of Allied Health, University of Limerick, Limerick, Ireland; 4 School of Pharmacy, Queen’s University Belfast, Belfast, UK; 5 School of Medicine, Dentistry and Biomedical Sciences, Queen’s University Belfast, Belfast, UK; 6 Center for Implementation Research, University of Arkansas for Medical Sciences, Little Rock, Arkansas, USA

**Keywords:** Delirium, Education, Health, Interdisciplinary education, Digital resource, Health profession students, Undergraduate education, Co-design, Co-production

## Abstract

**Background:**

Delirium is a common symptom of acute illness which is potentially avoidable with early recognition and intervention. Despite being a growing concern globally, delirium remains underdiagnosed and poorly reported, with limited understanding of effective delirium education for undergraduate health profession students. Digital resources could be an effective approach to improving professional knowledge of delirium, but studies utilising these with more than one profession are limited, and no evidence-based, interdisciplinary, digital delirium education resources are reported. This study aims to co-design and evaluate a digital resource for undergraduate health profession students across the island of Ireland to improve their ability to prevent, recognise, and manage delirium alongside interdisciplinary colleagues.

**Methods:**

Utilising a logic model, three workstreams have been identified. Workstream 1 will comprise three phases: (1) a systematic review identifying the format, methods, and content of existing digital delirium education interventions for health profession students, and their effect on knowledge, self-efficacy, and behavioural change; (2) focus groups with health profession students to determine awareness and experiences of delirium care; and (3) a Delphi survey informed by findings from the systematic review, focus groups, and input from the research team and expert reference group to identify resource priorities. Workstream 2 will involve the co-design of the digital resource through workshops (*n* = 4) with key stakeholders, including health profession students, professionals, and individuals with lived experience of delirium. Lastly, Workstream 3 will involve a mixed methods evaluation of the digital resource. Outcomes include changes to delirium knowledge and self-efficacy towards delirium care, and health profession students experience of using the resource.

**Discussion:**

Given the dearth of interdisciplinary educational resources on delirium for health profession students, a co-designed, interprofessional, digital education resource will be well-positioned to shape undergraduate delirium education. This research may enhance delirium education and the self-efficacy of future health professionals in providing delirium care, thereby improving practice and patients’ experiences and outcomes.

**Trial registration:**

Not applicable.

## Background

Delirium is an acute condition characterised by a rapid onset of cognitive decline with fluctuating symptoms. It can be triggered by a wide range of external stimuli such as surgery, infection, head injury, stroke, medication, substance withdrawal, deranged electrolytes and sleep deprivation [1]. Other features of delirium include altered consciousness, disorganised thoughts, disorientation, impaired memory, inattention, and paranoid delusions and hallucinations [2, 3]. Higher-risk groups include hospitalised older adults, children [4] and people requiring critical care; up to 80% of people admitted to the intensive care unit (ICU) experience delirium [5] and 30–80% of older adults experience delirium following major surgery [6]. Delirium can be profoundly distressing for patients, families and staff [1, 3]. Persistent exposure to delirium may indicate a symptom of diminished cognitive reserve, potentially heightening susceptibility to the development of dementia [7, 8]. There is some evidence that multicomponent, non-pharmacological interventions can reduce delirium incidence within in-patient hospital settings [9, 10].

Despite the association between delirium and increased mortality rates, extended hospital stays, and long-term impact on overall health, delirium remains underreported and underdiagnosed [5, 11, 12], however there is evidence of recent improvement [13]. Junior doctors in the United Kingdom (UK) and Ireland have demonstrated a lack of knowledge on how to effectively diagnose and manage delirium [14]. Furthermore, nurses also possess poor knowledge regarding delirium identification, with difficulties distinguishing between delirium, dementia, and depression reported [15, 16]. Therefore, to promote safe and effective care, healthcare professionals need to be equipped with the knowledge and ability to better identify, diagnose and manage delirium. Enhanced proficiency in this area allows for timely interventions and tailored care plans, emphasising the critical role of knowledgeable healthcare providers in optimizing outcomes for individuals with delirium.

Multidisciplinary approaches to delirium education, involving doctors, nurses, and pharmacists learning together, have been shown to be important in improving patient outcomes and learning experiences [17, 18]. Implementing multicomponent interventions, such as early mobilisation and family participation, have demonstrated benefits in reducing delirium incidence and duration [19]. For example, in a community hospital, the formation of an interprofessional consultative Delirium Team improved the prevention, detection, and management of delirium, with two-thirds of referred patients not requiring specialist consultation [20]. Multidisciplinary approaches to delirium education can therefore enhance patient outcomes and improve the overall learning experiences of healthcare professionals and have been recommended as part of clinical guidelines on the topic [2].

Interactive education such as role play, games, and simulation, have proven successful in improving qualified healthcare practitioners’ (e.g., nurses, doctors, physiotherapists) awareness of best practice associated with delirium care [21, 22]. However, pre-registration delirium education is inconsistent and varies considerably amongst health profession programmes, with approximately only 50% of UK universities providing medical education explicitly on delirium [23]. Further, in Ireland, limited studies have been identified to uncover what education, if any, is being provided on delirium. Previous studies have aimed to address such issues in undergraduate nursing studies through a face-to-face ‘delirium awareness’ program and podcast [24, 25] and through objective structured clinical examinations with medical students leading to improvements in knowledge and self-efficacy on identifying and managing delirium [26]. However, there remains a dearth of educational resources highlighting the importance of interdisciplinary teamwork in the identification and management of delirium.

Numerous delivery modes exist for educating healthcare professional students. Notably, the digital mode stands out for its success in interdisciplinary student education, fostering interprofessional socialisation, collaborative competencies [27], creative thinking [28], and serving as an effective platform for learning about telehealth [29]. Digital resources also provide a flexible mode of delivery, with participants able to access these in their own time. As not all universities have healthcare disciplines studying concurrently, the flexibility with digital resources may prove beneficial as face-to-face education with all disciplines may be impractical due to timetabling and placement timings. The aim of this study is therefore to co-design and evaluate a digital education resource to improve health profession students’ knowledge and self-efficacy in providing care to patients with delirium. Moreover, this study will involve:

I A systematic review to synthesise current evidence on digital delirium education interventions for health profession students.
II Focus groups with health profession students to understand their awareness and experiences of providing care to people with delirium.
III A series of questionnaires (Delphi study) to determine what key stakeholders perceive as the key education priorities associated with delirium.
IV Co-design of a digital education resource with health profession students, professionals, and those with lived experience of delirium.
V Evaluation of the digital resource to determine usability, perceived usefulness, the user experience, and preliminary efficacy in enhancing health profession students’ knowledge and self-efficacy for providing care to individuals with delirium.

## Methods

### Design

A logic model will be used to provide a systematic guide in the design of the study, including the decision on research outcomes and methods for data collection and analysis [30]. It is anticipated that the study objectives will be achieved through three key workstreams:

1. Generating theory.
2. Co-design of the digital resource.
3. Intervention evaluation.

An expert reference group (ERG) will be formed prior to study commencement, ensuring the study progresses as intended, that the protocol is adhered to, and to be involved when appropriate to support different elements of the study. The ERG will be comprised of representatives from Northern Ireland and the Republic of Ireland, with personal, professional, and educational knowledge and expertise on delirium. It is envisaged that this will include clinicians, former patients, family carers, policy makers, and educators.

### Workstream 1: generating theory

Workstream 1 (WS1) will inform the ‘inputs’ of the logic model. Comprised of three phases, WS1 aims to generate evidence and theory (systematic review and focus groups) and achieve consensus on the key education priorities associated with delirium education for health profession students (Delphi survey). The findings of WS1 will be utilised to inform the initial design and development of the digital education resource.

#### WS1 phase 1: systematic review

Phase 1 will involve a mixed methods systematic review to synthesise the current evidence on digital delirium education interventions for health profession students. The primary objective of this systematic review is to evaluate how pre-registration healthcare students are equipped to recognise, assess, and implement interventions for delirium prevention through digital or web-based educational interventions. Additionally, the review aims to inform the design of a future digital educational tool for delirium education. It encompasses both qualitative and quantitative studies to scrutinise the impact of existing digital or web-based delirium education programmes on the learning and practice of health professional students in higher education. The study seeks to explore the effectiveness of these digital education programmes, considering various factors such as educational context, professional backgrounds of students, and programme design. The review will also identify facilitators and barriers, assess measures of effect, undertake a critical appraisal assessment, and employ a mixed-methods synthesis approach. Potential subgroups within the broader cohort of health profession students may also be explored. Ultimately, this systematic review will provide comprehensive insights into digital education about delirium, shaping future educational strategies and contributing to enhanced patient care outcomes. A summary of the systematic review protocol is available via PROSPERO: (https://www.crd.york.ac.uk/prospero/display_record.php?RecordID=422411).

The findings of this review will help inform both WS2 and WS3. The review will be conducted in accordance with the Preferred Reporting Items for Systematic reviews and Meta-analyses (PRISMA) checklist [31, 32]. The following databases will be searched for eligible studies: CINAHL Complete, Medline, Embase, PsycINFO, Scopus, Web of Science, and Cochrane CENTRAL. Reference lists of relevant systematic reviews and scoping reviews will also be manually searched to ensure additional studies can be identified for inclusion. Studies eligible for inclusion must feature a digital or web-based education intervention on delirium for health profession students in tertiary/third level education, published between 2012 and 2023. The Joanna Briggs Institute Manual for Evidence Synthesis [32] will inform the integration of qualitative and quantitative data. Quality assessment will be conducted using the Crowe Critical Appraisal Tool (CCAT).

#### WS1 phase 2: focus groups with health profession students

Face-to-face focus groups will be conducted with health profession students to gain insight into their awareness and experiences of providing care to people with delirium. Approximately 32 health profession students will be recruited from two universities on the Island of Ireland (Queen’s University Belfast and University of Limerick). Participants will be recruited from a variety of health profession programmes, including student doctors, nurses, pharmacists and allied health professions. Students meeting the eligibility criteria will be contacted via email by their Director of Education or course lead, who will provide them with information about the study. These gatekeepers, serving in an independent capacity, will not be directly engaged in the research. Those students expressing interest in joining a focus group can reach out to the research team to receive an information sheet and details regarding the focus group schedule. Prior to participating in the focus group interview, all selected participants will be required to provide written consent. All participants will be reminded that their participation is voluntary and will not impact upon their course grade. Focus groups will be audio recorded and transcribed for analysis.

#### WS1 phase 3: Delphi survey

Guided by the findings from the systematic review and focus groups, a modified Delphi survey will be conducted to determine what key stakeholders perceive as the main priorities for delirium education for health profession students. It is anticipated that three rounds of surveys will be conducted with key stakeholders. Stakeholders will include individuals, organisations, and communities with a direct interest and/or expertise in health profession education on delirium. The items for the first round of the Delphi survey will be developed using empirical findings from Phases 1 and 2 of WS1, and through input of the research team and the ERG. Identification of delirium experts will be supported through engagement with various organisations including the British Geriatrics Society, the Royal College of Nursing, the All-Ireland Gerontological Nurses Association, the Irish Gerontological Society and other networks known to the research team.

The Delphi survey will be developed online using MS forms and emailed to those participants that have provided informed consent to be contacted via the professional networks. Participants will be asked to answer each item using a Likert scale, with each item requiring at least 75% agreement to proceed to the next round, or, in the case of the final round, be considered to have gained consensus. It is anticipated that 50 delirium experts (25 from each country) will be recruited to take part in the Delphi survey [33].

### Workstream 2: co-design of the digital education resource

Guided by the findings from WS1, workstream 2 (WS2) will focus on the co-design of the digital education resource with key stakeholders. The digital education intervention will form the ‘output’ of the logic model. Co-design of the digital education resource will be conducted with a co-design group, comprised of 15–18 health profession students representing medicine, nursing, pharmacy and allied health. Members of the co-design group will be recruited in the same way as noted in WS1, Phase 2. It is envisaged that four co-design workshops will be held in-person across a three-month period, two in Northern Ireland and two in the Republic of Ireland. To ensure development of the digital resource is evidence-based throughout, findings from WS1 will be incorporated throughout the co-design process, guiding the design, functionality, and key public health messages. The co-design approach proves invaluable for crafting education resources among student populations, as it actively involves them as end-users. This participatory method empowers students to prioritise and shape the content based on their preferences and specific learning needs, ensuring the resulting resources align closely with their educational requirements and enhance overall engagement [34, 35].

As part of the co-design process, students will be regarded as experts whose insights will inform critical decisions regarding the content of the digital education resource. One key aspect that will be explored is whether all healthcare professionals require uniform knowledge about delirium or if tailored sections specific to their roles are necessary. For instance, doctors may prioritise understanding the pathophysiology of delirium, while pharmacists might focus on medications and polypharmacy, and nurses may emphasise initial symptom recognition. This iterative approach acknowledges the diverse learning needs within healthcare disciplines and recognises that preferences may vary. By integrating empirical evidence from workstream 1, including a systematic review, qualitative focus groups, Delphi survey, and the co-design methodology itself, the development process will remain evidence-based and responsive to the evolving needs of the end-users.

### Workstream 3: evaluation

WS3 will involve the mixed methods evaluation of the digital resource with health profession students through two phases, producing the ‘outcomes’ of the logic model. This process will be guided by the technology acceptance model (TAM) [36] and Proctor et al.’s [37] taxonomy of implementation outcomes. Originating in 1986, the TAM aids in understanding predictors of human behaviour regarding acceptance or rejection of technology through two variables that may affect the adoption of digital technology: 1) perceived usefulness and 2) perceived ease of use.

These variables, and students’ usage patterns, will be assessed as part of the intervention evaluation. The components of Proctor et al.’s taxonomy of implementation outcomes that will underpin the evaluation are:

- Acceptability
  - *The perception among stakeholders that the given treatment, service, practice, or innovation is agreeable, palatable, or satisfactory.*
- Adoption
  - *The intention, initial decision, or action to try or employ an innovation or evidence-based practice (uptake)*.
- Appropriateness
  - *The perceived fit, relevance, or compatibility of the innovation for a given practice setting, provider, or consumer; and/or perceived fit of the innovation to address a particular issue or problem.*

#### WS3 phase 1: health profession students’ knowledge, self-efficacy, and usability

Phase 1 will involve the assessment of health profession students’ knowledge and self-efficacy of providing care to those with delirium through a pre- and post-test questionnaire. Two questionnaires, the 35-item Delirium Knowledge Questionnaire (DKQ) [38, 39] and a 3-item questionnaire on self-efficacy towards recognising and providing care to those with delirium [24, 25]. Questionnaires will be delivered to participants at baseline and four weeks after the delivery of the intervention.

Approximately 300 health profession students from both Queen’s University Belfast and University of Limerick will be recruited through convenience sampling in the same matter noted in WS1, Phase 2 and WS2. Students will be provided with an opportunity to an online information sheet detailing the study and how to use the resource. A series of questions will be displayed to gain consent, with access to the questionnaire granted only once consent is provided. Usability of the digital resource will be assessed through the validated 10-item questionnaire, the ‘System Usability Scale’ [40] provided post-test. Lastly, participants will be provided with two ‘open text’ questions to facilitate additional comments on what students liked or disliked about the resource, and to suggest ideas for future dissemination or testing activities. Internal analytics such as user engagement and time spent on the resource will be used to further determine usability and acceptability.

#### WS3 phase 2: perceived ease of use and usefulness of the digital resource

The second and final phase of the evaluation will aim to uncover health profession students’ perceptions regarding the ease of use and usefulness of the digital resource. Four focus groups will be conducted with health profession students (*n* = 32) who have previously used the resource. These participants will be asked to provide consent to be contacted for this part of the evaluation after they complete WS3, Phase 1. Dependant on data saturation, the number of participants may be either increased or decreased. Focus group questions will be developed by the research team, aligned with the RE-AIM (Reach, Effectiveness, Adoption, Implementation, Maintenance) framework [41] and Proctor et al.’s taxonomy on implementation outcomes [37]. Students will be asked to share suggestions on approaches for the resource to *reach* a wider student audience, how they would identify the *effectiveness* of the resource, ways in which the resource could be *adopted* and *implemented* by healthcare and educational institutions, and how it should be *maintained* over its lifetime.

### Data analysis

#### Qualitative analysis

Audio recordings and notes gathered through the focus groups in WS1 and WS3 will be transcribed verbatim and uploaded to NVivo 12 management software for analysis, along with the ‘open text’ comments from the post-test questionnaire (WS3) and those gathered during the co-design workshops (WS2). Qualitative data will be coded and analysed using thematic or narrative synthesis (systematic review), constant comparative analysis (WS1 focus groups), and thematic or directed qualitative content analysis (WS2 and WS3 focus groups).

#### Quantitative analysis

This will involve quantitative data from the review’s data extraction form (WS1) and evaluation data from the pre-post questionnaires (WS3). Quantitative data analysis will be conducted using SPSS v.26. Descriptive statistics will be employed to report on results of the Delphi study, participant demographics, and internal analytics. Paired t-tests will be used to assess changes to health profession students’ knowledge of delirium and self-efficacy to determine preliminary efficacy. Additionally, demographic details will be collected to gauge which participants engage with the resource (providing data on its reach).

## Discussion

The implementation of an online platform for the delivery of the co-designed digital education resource will not only address the pressing need for interprofessional education on delirium care but also has advantages associated with digital learning environments. Despite a scarcity of literature specifically addressing interprofessional digital education resources on delirium and their impact on delirium knowledge, self-efficacy, and healthcare practice, existing studies examining the effects of digital education interventions on professional knowledge and self-efficacy offer important findings. Research has shown that digital education interventions can significantly enhance learning outcomes for both health profession students and registered professionals, providing an engaging and interactive learning experience [42–47]. By adopting an immersive approach with real-time feedback, digital interventions have the potential to boost students’ confidence and self-efficacy in their knowledge and skills [44–46].

One of the key advantages of digital interventions is their superior accessibility compared to traditional classroom-based teaching methods, particularly in the context of interdisciplinary education. This accessibility ensures that students from various healthcare disciplines, with differing schedules and logistical constraints, can access essential information about delirium care at their own convenience [48]. Moreover, the asynchronous nature of digital resources facilitates flexible learning, allowing students to progress through the material at their own pace and revisit key concepts as needed. This flexibility is particularly advantageous given the complexities of coordinating educational sessions involving nurses, doctors, and pharmacists, who may have disparate timetables and program requirements.

While the e-resource will serve as a valuable adjunct to traditional educational methods, it is essential to recognise that it will not be designed to replace simulation, practice-based learning, or face-to-face teaching that may already be in place within a healthcare professional programme. Rather, the aim is to complement existing educational practices by providing a comprehensive overview of delirium care and promoting interdisciplinary collaboration among healthcare professionals.

### Limitations and challenges

There are several limitations to consider in this study. First, there is a risk of response bias through the pre-post questionnaires (WS3) due to self-reporting which may not reflect the true impact of the digital resource on knowledge and self-efficacy. However, the utilisation of validated questionnaires should minimise this limitation. Second, as the resource will be developed in the context of health profession students on the island of Ireland, the generalisability of the resource to other contexts and populations of health profession students may pose a limitation. However, similar standards and competencies are expected for undergraduate health profession programmes nationally and internationally, it is expected that the digital resource will have reach and the potential to be adapted for different cultural contexts.

Lastly, there are potential challenges in the recruitment and retention of health profession students throughout different stages of their education programme. Thus, findings of this study may not be representative of the learning needs and preferences of all health profession students. However, through integrating local, national, and international evidence, and gaining insight from experts and those with lived experience of delirium, it is expected that the resource will be an engaging and evidenced-based resource that is acceptable to end-users.

## Data Availability

No datasets were generated or analysed during the current study.
